# Preventing Binge Eating with Deep Brain Stimulation – Can Compulsive Eating be Switched Off?

**DOI:** 10.3389/fpsyt.2013.00168

**Published:** 2013-12-13

**Authors:** Sarah I. Martire, Dominic M. D. Tran, Amy C. Reichelt

**Affiliations:** ^1^School of Psychology, University of New South Wales, Sydney, NSW, Australia; ^2^School of Medical Sciences, University of New South Wales, Sydney, NSW, Australia

**Keywords:** binge eating, deep brain stimulation, nucleus accumbens shell, dopamine, opioids, addiction, obesity

Reward-seeking behaviors, including palatable food consumption, are underpinned by activation of mesocorticolimbic dopamine (DA) neurocircuitry, resulting in extracellular release of DA in the nucleus accumbens (NAc) (1). Dysregulation of the mesocorticolimbic DA system is proposed to underpin binge eating – the compulsive consumption of palatable food within a short time period. Intermittent access to palatable food can induce addiction-like behaviors including binging, withdrawal, and cravings, producing persistent DA increases within the NAc shell (NAcSh), similar to drug self-administration (1).

Modulation of the neuronal responsiveness to the rewarding properties of food by NAcSh deep brain stimulation (DBS) provides a potential treatment for maladaptive behaviors including drug addiction. Halpern et al. (2) explored DBS as a prospective intervention for binge eating; contributing to literature suggesting NAcSh DBS can attenuate drug addiction (3, 4). NAcSh DBS decreased binge eating a palatable high fat food, possibly modulated by DA. Systemic administration of the DA D2 receptor antagonist raclopride reversed the hypophagic effect of DBS, suggesting that DBS modulated the palatability of foods via DAergic neurocircuitry. However, the interpretation of this study was restricted to dopaminergic neurotransmission, and the animal model of binging presented has limitations in the preclinical recapitulation of human behavior.

It was proposed that DBS decreased the palatability of binge-evoking foods. However, a distinction between “liking” and “wanting” rewards has been made which can be extended to palatable foods (5). DA transmission in the NAc is attributed to “wanting,” which is the *desire* to acquire a reward, rather than “liking,” which is the *hedonic (pleasurable) impact* of rewards (6). Elevated extracellular NAc DA has no effect on hedonic responding for sucrose, but increased lever presses indicative of enhanced wanting (7), supporting the proposal that DA is important in the mediation of reward-related cues to trigger “wanting,” as opposed to the hedonic appraisal of foods.

The observed DBS modulation of palatability suggests effects may extend to other reward-related neurotransmitter systems. The NAcSh contains a large concentration of μ-opioid receptors, which influence appetitive behaviors (8), and a specific “hotspot” for μ-opioid hedonic enhancement, into which microinjection of the μ-opioid agonist DAMGO increased hedonic reactivity to intraoral infusions of sucrose (8). To establish the role of the μ-opioid system in NAcSh DBS, it should be investigated as to whether μ-opioid receptor ligands reinstate binge eating. Additionally, whether NAcSh DBS modulates taste reactivity (e.g., orofacial reactions, licking microstructure), indicative of the hedonic value of a food, should be established. Together, this may offer further insight into the neural mechanisms underpinning DBS and binge eating with respect to modulation of the hedonic or incentive value of foods.

Whilst it remains unclear whether DBS modulated hedonic or incentive mechanisms; it remains possible that the inherently rewarding properties of DBS reduced responsiveness to palatable foods by manipulating reward thresholds. In case studies of unrestricted human electrical brain self-stimulation, subjects reported a “desire” to self-stimulate (9) and this may regulate rewarding behavior performance.

The raclopride-induced reinstatement of binge eating was central to the proposal that DA D2 receptors regulate binge eating. However, raclopride can modulate appetite and reward-related behaviors (10). Consequently, the reinstatement of binge eating may be due to raclopride-induced hyperphagia, supported by increased consumption following raclopride administration on DBS-off test days.

The modulatory effect of DBS may extend beyond the NAc to regions involved in motivated behaviors. Although the mechanisms of DBS are not yet fully understood, it is known that effects extend to neurocircuitry associated with the target region (11). In a recent fMRI study, NAcSh DBS modulated activity within the prefrontal cortex, cingulate, and insula (12), regions functionally involved in cue-induced cravings and responsiveness to food-associated stimuli. The DBS-increased responsiveness of regions involved in cognitive control may therefore override compulsive behaviors underpinning addictive behaviors.

Animal models are important in understanding neural mechanisms, however may not capture emotional components of binge eating. The mouse model utilized by Halpern et al. allows limited access to high fat pellets until “binge like” behaviors, defined as >25% of daily caloric intake consumed within 1 h, are produced. This model is suggested to provide preclinical support for the use of DBS in controlling aberrant binge eating. Limited access to fat evokes binging (13, 14) and increases NAc DA levels (15), however withdrawal-like behaviors have not been observed (16). Limited daily access to sugar results in behavioral adaptations such as escalation of consumption (17) and withdrawal (18), as well as altered DA and μ-opioid binding profiles (19). Cycling access to palatable foods may provide a more accurate depiction of binge eating disorder, provoking binging in rats when not in a state of hunger and following physical stressor exposure (20, 21). Thus, DBS should be applied in models capturing physiological and behavioral aspects of binging, such as limited sugar access, or more translational models, such as cycling palatable food access.

Halpern and colleagues provide a preliminary insight into the use of DBS to treat binge eating, however their demonstration of the efficacy of DBS is not entirely compelling. The effects of chronic DBS are limited as high fat diet consumption increased across the course of continued NAcSh DBS. Furthermore, chronic DBS had negligible effects on total calories consumed, and acute use of DBS to prevent binging may not be efficacious in clinical settings if effects are transient. Consumption of food is fundamental for life, thus modulating maladaptive ingestive behaviors can be considered more complex than drug self-administration due to continuing physiological requirements. Further investigations employing DBS in models more applicable to human binge eating disorder are needed to elucidate DBS as a therapeutic option. Presently, Halpern et al.’s findings cannot be extended to clinical settings and more rigorous preclinical experimental investigations are required.
